# Reducing the Cost of the Diagnostic Odyssey in Early Onset Epileptic Encephalopathies

**DOI:** 10.1155/2016/6421039

**Published:** 2016-05-08

**Authors:** Charuta Joshi, Diana L. Kolbe, M. Adela Mansilla, Sara O. Mason, Richard J. H. Smith, Colleen A. Campbell

**Affiliations:** ^1^Division of Pediatric Neurology, University of Iowa Children's Hospital, Iowa City, IA 52242, USA; ^2^Iowa Institute of Human Genetics, University of Iowa, Carver College of Medicine, Iowa City, IA 52242, USA

## Abstract

Whole exome sequencing (WES) has revolutionized the way we think about and diagnose epileptic encephalopathies. Multiple recent review articles discuss the benefits of WES and suggest various algorithms to follow for determining the etiology of epileptic encephalopathies. Incorporation of WES in these algorithms is leading to the discovery of new genetic diagnoses of early onset epileptic encephalopathies (EOEEs) at a rapid rate; however, WES is not yet a universally utilized diagnostic tool. Clinical WES may be underutilized due to provider discomfort in ordering the test or perceived costliness. At our hospital WES is not routinely performed for patients with EOEE due to limited insurance reimbursement. In fact for any patient with noncommercial insurance (Medicaid) the institution does not allow sending out WES as this is not “established”/“proven to be highly useful and cost effective”/“approved test” in patients with epilepsy. Recently, we performed WES on four patients from three families and identified novel mutations in known epilepsy genes in all four cases. These patients had State Medicaid as their insurance carrier and were followed up for several years for EOEE while being worked up using the traditional/approved testing methods. Following a recently proposed diagnostic pathway, we analyzed the cost savings (US dollars) that could be accrued if WES was performed earlier in the diagnostic odyssey. This is the first publication that addresses the dollar cost of traditional testing in EOEE as performed in these four cases versus WES and the potential cost savings.

## 1. Introduction

Early onset epileptic encephalopathies (EOEEs) encompass a wide variety of early onset seizure disorders invariably associated with difficult-to-treat seizures, developmental delay or stagnation, poor prognosis, and uncertainty regarding standard treatment approaches [1]. Traditionally, a stepwise investigative approach (henceforth referred to as traditional method) was not only the most logical but also the most economical approach to diagnoses made on the basis of phenotype specificity. Traditional methods of testing are heavily weighted towards inherited metabolic disorders as causes of EOEE. However, it has become increasingly apparent that many cases of EOEE have a genetic basis. For most EOEE cases a good genotype/phenotype correlation is not yet established. Furthermore, the causative genes involved include broader abnormalities than the typical ion channel abnormalities that epileptologists are used to thinking about when thinking through EOEE. Genetic diagnosis at an earlier stage is now possible using next generation sequencing techniques such as targeted gene panels or whole exome sequencing (WES). There is limited insurance coverage for WES at this time. In fact although many hospitals in the United States will allow testing for genetic panels through commercially available laboratories (like, e.g., GeneDx and Athena Diagnostics) even major epilepsy centers do not allow routine testing for WES forcing clinicians to continue to approach the diagnosis of EOEEs using traditional methods. In this day and age of increasing healthcare costs it is common practice to look at the tests ordered by physicians in terms of their utility in changing patient management and diagnostic solve rate. This guides the establishment of hospital policies in what types of testing can be routinely ordered in an outpatient setting. For patients that do not have commercial insurance, the hospital is liable for “eating up” the cost for any noncovered test. For any patient with noncommercial insurance (Medicaid) our institution does not allow sending out WES as this is not “established”/“proven to be highly useful and cost effective”/“approved test” in patients with epilepsy.

Most patients with an epileptic encephalopathy have an MRI, serum amino acids, urine organic acids, homocysteine, ammonia, cerebrospinal fluid analysis, chromosomal microarray (CMA), array comparative genomic hybridization (aCGH), and blood chemistries in a variable combination as the first tier of investigation. Frequently, these tests are followed by more invasive second- and third-tier studies that include gene panels or skin/muscle biopsy. Insurance companies routinely approve these first-, second-, and even third-tier tests, despite the fact that, with the exception of MRI and aCGH or CMA, the diagnostic yield is <5% for additional metabolic tests or tissue biopsies in EOEE [2, 3]. A recent paper outlines the algorithm used and diagnostic yield of various tests used at The Hospital for Sick Children in Toronto, Canada, for investigation of EOEE [2].

The corresponding author C. Joshi had been caring for 4 patients with EOEE with noncommercial insurance for several years. After exhaustive searching for etiology for EOEE that included gene panels for epilepsy, it became apparent that the best possible solution would be WES. The patients were tested in a clinical diagnostic lab through a research protocol as a proof of principle study. All patients had a definitive diagnosis through trio WES, and results were confirmed in a CLIA lab. In an attempt to further assess “the value” of WES in EOEE, we analyze the dollar cost of WES offered through the Iowa Institute of Human Genetics (IIHG) against traditional methods of investigation for EOEE in these four patients. As none of these patients had commercial insurance WES was offered to them at no cost on a research basis.

## 2. Methods

### 2.1. Case Reports

We present three short vignettes of four children with EOEE in whom we performed retrospective chart review and definitively diagnosed EOEE by WES. Prior to trio WES, all four children had undergone extensive studies over many years using traditional tests (Table 1). The diagnostic results are summarized in Table 2. These four patients were selected for this cost analysis study since their diagnostic odysseys had come to an end upon receiving a genetic etiology for EOEE and no further diagnostic testing was necessary.

#### 2.1.1. Case 1 and Case 2 (C1, C2)

**Figure 1:**
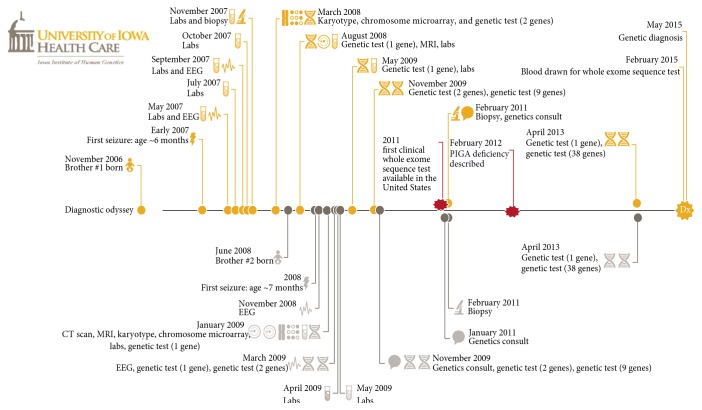
A timeline indicating the 8-year diagnostic odyssey for patients C1 and C2.

Patient C1 is a 7-year-old nondysmorphic boy with medically intractable epilepsy with onset at age 4 months. He presented with nonfebrile status epilepticus repeatedly between ages of 4 and 8 months and failed five antiepilepsy medications (AEDs). He stopped seizing altogether after starting ketogenic diet (KD) but remained significantly developmentally delayed and currently has autistic features with a nonverbal, nonambulatory phenotype. He had extensive genetic and metabolic testing using traditional methods (Table 1). Family history was significant for an older brother, C2, who presented in infancy with repeated status, had regressed, and was 18 months old, severely developmentally delayed, nonverbal, and nonambulatory when he came to our attention. With the proband's success with KD, his older brother was switched to KD monotherapy; he has had no further seizures on a KD. Given the high probability of a genetic cause, WES was performed and a single variant, NM_002641:c.A535T:p.N179Y, in the gene* PIGA* was identified in both brothers and their mother, consistent with the diagnosis of X-linked recessive phosphatidylinositol glycosylation protein A deficiency (PIGA deficiency) [4]. The length of the diagnostic odyssey in the older brother spanned eight years (Figure 1, Table 3).

#### 2.1.2. Case 3 (C3)

**Figure 2:**
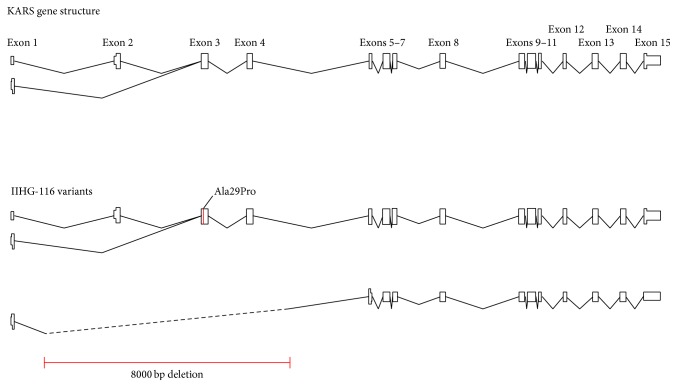
*KARS* has two isoforms, one retained in the cytoplasm and one transported to the mitochondria. A mitochondrial targeting sequence spans exons 2 and 3; the cytoplasmic isoform skips exon 2 and is not transported. Case 3 has two inherited mutations that disrupt the gene. An Ala29Pro missense mutation alters the end of the mitochondrial targeting sequence; we predict that it is not transported, but the cytoplasmic isoform is still functional. The other allele has an approximately 8 kb deletion spanning exons 2–4, resulting in a frameshift and early termination.

Patient is an 18-month-old dysmorphic young boy who came to our attention at age 72 days due to nystagmus, failure to thrive, inability to fixate, microcephaly, hypertonicity, and extreme irritability. By age 6 months, he had developed modified hypsarrhythmia on his EEG, which was performed to evaluate abnormal eye movements. Treatment with levetiracetam seemed to calm him down. Although he never developed epileptic spasms, his EEG improved by age 10 months to focal epileptiform discharges. Once again, an extensive genetic metabolic investigation (Table 1) failed to reveal a cause of his symptoms. WES was done at age 14 months due to parents' desire to have more children. Two variants, a paternally inherited NM_005548:c.85G>C, p.Ala29Pro variant, and a maternally inherited 7601-base pair deletion at chr16:75672800-75680400 on 16q23.1, in the Lysyl-tRNA Synthetase (*KARS*) gene were identified, consistent with autosomal recessive inheritance of KARS-mediated disease (Figure 2) [5].

#### 2.1.3. Case 4 (C4)

Patient is a 26-month-old nondysmorphic female with a history of tonic spasms starting at age 3 months. She failed six AEDs by age 12 months. Past medical history was significant for hypotonia noted at 2 days of age, as well as abnormal posturing that was deemed to not be seizures as an interictal EEG was normal. When seen at our center at age 16 months, she had an extensive genetic and metabolic workup (Table 1). Notable features on exam were severe hypotonia, lack of fix or follow, lack of spontaneous movements, and frequent myoclonic jerks/tonic jerks of arms and legs. She was fed via G-tube and slept up to 22 hours of the day. She had one healthy older sister and another sister with absence of the corpus callosum. WES sequencing was performed and a* de novo* dynamin 1 (*DNM1*) mutation, NM_001005336:c.1075G>C, p.Gly359Arg, was found as the cause of her early infantile epileptic encephalopathy [6] and was not present in either parent.

### 2.2. Categorization of Patient Investigation

We have adapted The Hospital for Sick Children diagnostic algorithm [2] to categorize investigations in this report. Tier 1 tests include clinical assessment, detailed neurological examination, brain MRI, response to pyridoxine where clinically indicated, chromosomal microarray, plasma amino acids, urine organic acids, homocysteine, cerebrospinal fluid analysis for amino acids, and glucose in cerebrospinal fluid. The expected diagnostic yield in the Sick Kids Cohort from the sum of all these tests was ~15%. Tier 2 tests include epilepsy gene panels, which increased the diagnostic yield to up to 50% of EOEE patients. Tier 3 tests include muscle and skin biopsies and add about 1% to the diagnostic yield.

### 2.3. Cost Analysis

Actual dollar values charged for these tests were abstracted from hospital billing using the electronic medical record which was available to us after July 2009. Any charges prior to July 2009 were hand searched and if not found, present day charges were applied. The cost analysis does not include additional consultations with other medical specialists, such as ophthalmologists and geneticists. Any testing was done as part of clinical care (electroencephalograms (EEGs) or testing for vitamins and minerals in patients on ketogenic diet or serial MRI looking for cortical dysplasia in epileptic patients was not counted here).

### 2.4. Whole Exome Sequencing

Although the patients were enrolled on research consent, the trio whole exome sequencing was performed following a clinical testing protocol in a CLIA accredited lab. 3 *μ*g DNA was prepared from peripheral blood from each patient and their parents and processed for WES using SureSelectXT Human All Exon V4 as recommended. WES was completed on indexed samples that were pooled and sequenced using paired-end chemistry over one or two lanes of the HiSeq 2500 platform (Illumina, San Diego, CA). Data processing was completed as described, routing the primary FASTQ output files into an optimized Galaxy Analysis pipeline. Sequences were aligned to hg19 using BWA-MEM; variants were called using GATK; and the variant list was annotated with Annovar to list gene, mutation type, conservation, and MAF [7–14].

Symptom-guided analysis was utilized for variant filtering. Candidate genes and inheritance models were selected based on the clinical history provided by the ordering physician, pedigree analysis, and genes known to cause epilepsy using OMIM (http://www.ncbi.nlm.nih.gov/omim), PubMed (http://www.ncbi.nlm.nih.gov/pubmed), GeneReviews (https://www.genetests.org/resources/genereviews.php), and PhenoTips (http://phenotips.org/). The IIHG diagnostic lab routinely reports candidate gene regions with sequencing coverage less than 10x to the ordering physician for genes on the symptom-guided analysis gene list. Based on reported family history, multiple inheritance models were considered. Variants were classified as pathogenic by ACMG guidelines which take into account variant frequency and pathogenicity prediction scores [15]. All results were discussed by the Iowa Institute of Human Genetics Clinical Exome Interpretation Team, as well as the ordering physician. Identified variants considered causative were confirmed by Sanger sequencing (Table 3) in the IIHG CLIA accredited lab. To confirm the* KARS* deletion, NM_005548 p.Asn21_Gly130del, a PCR and agarose gel based assay was designed with primers flanking the deletion and PCR product was verified in an agarose gel. In addition, the deletion PCR products were Sanger sequenced to confirm the deletion breakpoints. As exome testing was performed in a clinical diagnostic lab, the ordering physician received results report that included coverage on all candidate genes.

## 3. Results

Our patients had an average of 20.25 traditional tests (range 17–24) (Table 1) until WES was performed and led to definitive diagnosis (Table 2). The time in years spent on this diagnostic odyssey ranged from 1.4 to 8.2 years, Table 3. As shown in Table 4, patient C2 underwent muscle and nerve biopsy, the cost of which alone was $10,000; C1 underwent serial single gene testing amounting to $35,483; C3 underwent microcephaly gene testing; and C4 underwent mitochondrial gene testing at an outside hospital. The total cost of these traditional tests ranged from $9,015 to $35,483. The cost of a WES trio is $6100. Since this testing was performed in a clinical lab, the turnaround time for the WES result was 12 weeks, Table 3.

## 4. Discussion

### 4.1. Types and Number of Tests Performed in EOEE

Patients with EOEE and other intractable epilepsies are referred to a tertiary epilepsy center for workup and possible definitive therapy. The burden of making the correct diagnosis, thereby bringing closure, offering a possible definitive treatment to salvage development in a young brain, and offering possible genetic counseling therefore falls on the pediatric epileptologist and members of their multidisciplinary team. In a recent study looking at the etiological yield of patients undergoing testing for infantile spasms, only 4.5% additional patients were found to have a definitive diagnosis once a physical examination and MRI were negative [3]. In that same paper, the yield of aCGH and genetic panels combined was >40% despite the fact that only 26 patients were evaluated with an epilepsy gene panel in the group without an obvious cause for spasms at diagnosis. The four cases presented in this paper illustrate the pitfalls of a traditional diagnostic assessment. The length of the diagnostic odyssey in patients C3 and C4 was much shorter as they did not have stepwise single gene testing or further invasive testing. After a negative infantile epilepsy gene panel test, WES testing was completed. It should be noted that, for these four patients, three of the four patients (C1, C3, and C4) had at least one gene panel test prior to exome testing that failed to yield a diagnostic result. The fourth patient (C2) is the brother of one of the probands so panel testing was not performed as it had been previously performed for his brother. Furthermore, two of the patients (C1, C3) had two or more gene panels that failed to yield a diagnostic result. The failure of gene panels to yield a diagnostic result in EOEE is not surprising given the rapid advancement of novel gene discovery in epilepsy research. It is important for clinicians to select clinical labs that report gene coverage for WES tests as not all known epilepsy genes will be fully covered. Although this study was done on a research basis, the lab was selected based on their clinical testing protocol and test report which includes all candidate gene regions that are not sequenced to at least 10x coverage and a 12-week turnaround time.

Over the last five years, several articles have explored the value of genetic testing in EOEE [16, 17]. In a retrospective cohort of 110 patients with EOEE [2], 28% patients received a genetic diagnosis. In only 7% was the EOEE due to an inherited metabolic disorder where all patients had a clinical or biochemical feature suggestive of a metabolic disorder. 12.7% of the 110 patients were diagnosed with gene panel testing or aCGH. None of these patients had WES. In the patients with nonmetabolic EOEE, only 17% had some clinical feature suggesting their underlying disease. These authors thus recommend next generation sequencing including gene panels and clinical WES for the identification of genetic causes of EOEE in patients with normal first-line biochemical investigations, normal aCGH, no seizure response to pyridoxine or pyridoxal-5-phosphate, and no recognizable syndrome or characteristic brain MRI changes that point to a specific genetic disorder.

### 4.2. Cost of the Diagnostic Odyssey

Diagnostic odysseys have three cost components: cost of time lost to the patient/family, quality of life, and monetary cost. We attempted to highlight some of these in this study. Our findings of the total additional (additional after tier 1 tests) monetary cost ranged from $9,015 in C4 to $35,483 in C1. If we were to subtract the cost of every single gene test from C1's cost analysis (cost for batten screen,* CDKL5*,* SCN1A* gene sequencing, Rett gene sequencing,* ATP7A* for Menkes disease, and X-linked mental retardation-sequencing of a panel of 7 genes), the excess cost would have still been $13,055. In terms of the time spent since the first test sent, Rett gene sequencing in March 2009 to* ATP7A* testing for Menkes sent in April 2013, we could have saved five years. In addition, although the testing in this study was performed free of charge on a research basis, the cost of a clinical trio WES test at the IIHG is $6100. The cost of current epilepsy gene panels ranges from $1500 to $6000 for testing only the proband (https://www.nextgxdx.com/).

In an article detailing the effectiveness of WES [18] and whole genome sequencing (WGS) in 119 children with a wide variety of acute or chronic neurodevelopmental delay, patients were noted to have spent an average of $19,000 on diagnostic tests other than WES/WGS. Patients included in that study were accrued from both the acute or chronic setting and had a wide variety of disorders including mental retardation, autism, seizures, or low tone. Average age of starting investigations in these patients was 6 months and average age at diagnosis was 95 months; thus, average length for their diagnostic odyssey was 8 years. This is longer than the diagnostic odysseys seen in our cases and may be representative of the “nonacute” nature of the other cohort.

Although in a small case series as ours cost of changes to quality of life cannot be objectively measured, all of our patients uniformly expressed gratitude due to the closure the diagnosis allowed and also a feeling of allayed guilt in the parents about the EOEE somehow being their fault. Larger formal studies on the cost to quality of life should be performed in the future.

The diagnostic yield of these four research based patients was 100%. It is common knowledge that the diagnostic yield of WES varies widely from 11 to 67% depending on the number of cases tested and the clinical indication [17, 19–21]. Gene panels are dependent on identifying abnormalities in “candidate genes.” However the scope and nature of “candidate genes” being implicated in EOEE are changing at a rapid rate. It is important to note that the causative gene in all four patients was described within the last three years and thereby was not included on any of the targeted gene panels previously tested (Figure 1, Tables 2 and 4).

As healthcare costs increase, many tests have to be preapproved by payers if reimbursement is to be obtained. This is especially true for tests that are considered “investigational” or do not have an established cost-benefit ratio. Lack of reimbursement still remains a major hurdle to implementation of tests like WES although WES was first available clinically in 2011. Nevertheless, our results clearly demonstrate the diagnostic power and cost savings of WES in patients with epileptic encephalopathy. The ability to identify a genetic etiology early saves healthcare dollars and guides treatment and has significant implications for the family. For example, in these cases genetic diagnosis obviated the need for additional clinical tests such as MRIs and in Cases 1 and 2 prompted a change in the physical therapy regimen. Genetic counseling for recurrence risk and disease prognosis was also critical to all three of these families for family planning.

Our paper has the obvious limitations of being a small retrospective case series with practice trends limited to one physician's experience. It is possible that some of the tests done at outside hospitals could have been missed in this analysis. We were also not able to abstract the actual cost of tests done before July 2009 as those charges are not saved by our hospital billing department. However, most genetic tests have a lower cost with time and hence we feel that applying present day charges is still a reasonable way to assess the cost of the few tests that were done prior to July 2009. Since we compared the cost of present day WES against other tests, it is also still meaningful to assess what present day charges might have been for the other tests in the event a physician were to continue to follow traditional methods of diagnosis in EOEE. Another limitation is that the nonobjectively assessed costs of the diagnostic odyssey (psychological cost of not having closure) could not be calculated.

We believe that WES with a multidisciplinary team approach and decision logic tree can help reduce all of the costs associated with a diagnostic odyssey by reducing the time to diagnosis; reducing the amount of uncertainty and stress for families by not knowing a diagnosis; and reducing the amount of healthcare dollars spent.

## 5. Conclusion

There is much phenotypic overlap in EOEE and looking for a particular distinguishing phenotype is often futile. Epilepsy gene panels and aCGH are a good way to analyze EOEE. However, the concept of “candidate genes” causing EOEE is rapidly changing, thus making the yield of epilepsy panels variable. In addition, panels vary in the number of genes tested and also by phenotypic categorization of patients ranging from 35 to 327 genes depending on the commercial company utilized. The phenotypic overlap in patients with EOEE could result in a wide range of solve rates.

When considering EOEE and implications for closure, future development, and genetic counseling, after performing a basic metabolic workup, aCGH, and gene panel testing specific for EOEE, money is more wisely spent on WES than other traditionally accepted “fishing expeditions” that inevitably are focused on inherited disorders of metabolism. This is the first paper to focus on cost savings solely in the population of EOEE. Greater numbers of patients need to be prospectively studied to see whether the cost savings of early WES are reproducible across large number of patients with EOEE across many centers.

## Figures and Tables

**Table 1:** Summary of all diagnostic studies performed on 4 patients with EOEE.

Test	C1	C2	C3	C4
Routine blood chemistry and complete blood count	√	√	√	√
Plasma amino acids	√	√	√	√
Urine organic acids	√	√	√	√
Urine creatine	√		√	
Urine alpha amino adipic semialdehyde			√	
Acylglycine/acyl carnitine profile	√	√	√	√
Very long chain fatty acids	√		√	
Serum peroxisomal panel				√
Vitamin/mineral assays	√	√	√	√
Carbohydrate deficient transferrin	√	√		√
MRI brain	√	√	√	√
Cerebrospinal fluid for routine testing	√		√	√
Cerebrospinal fluid for amino acids	√			
Cerebrospinal fluid neurotransmitters	√			√
Chromosomal microarray	√	√	√	√
Karyotype	√		√	
Molecular FISH		√		
Angelman/Prader Willi genetic testing		√	√	√
Epilepsy panel	√		√	√
X-linked mental retardation panel	√			
X-linked microcephaly panel			√	
Dual genome panel		√		√
Leukocyte lysosomal assay	√	√	√	
Rett syndrome	√			
SCN1A	√			
CDKL5	√			
ATP7A deletion/duplication and sequencing	√	√		
SLC9A testing		√		
Urine organic acids	√	√	√	
Urine sialic acid	√	√		
Urine creatine analysis		√		
Mitochondrial analysis (various methods)		√		√
Muscle biopsy		√		
Nerve biopsy		√		
Skin biopsy		√		
EEG	√	√	√	√
EMG				√

**Table 2:** Genetic results for patients in this study from whole exome sequencing.

Patient	Causative variant(s)	Causative gene	OMIM number	Inheritance	Reference
C1	NM_002641:c.A535T:p.N179Y	*PIGA*	311770	X-linked recessive	Johnston et al. 2012 [4]
C2	NM_002641:c.A535T:p.N179Y	*PIGA*	311770	X-linked recessive	Johnston et al. 2012 [4]
C3	NM_005548:c.85G>C, p.Ala29Pro; and chr16:75672800-75680400del	*KARS*	601421	Autosomal recessive	McMillan et al. 2015 [5]
C4	NM_001005336:c.1075G>C, p.Gly359Arg	*DNM1*	602377	Autosomal dominant	EuroEPINOMICS-RES Consortium et al. 2014 [6]

**Table 3:** Length of diagnostic odyssey for four patients with EOEE.

Patient	Age at first presentation of symptoms	Age when blood was drawn for WES	Age at final genetic diagnosis	Length of diagnostic odyssey	Change in medical management or familial impact
C1	4 months	6.7 years	7.0 years	6.3 years	Change in physical therapy

C2	4 months	8.3 years	8.6 years	8.2 years	Change in physical therapy

C3	72 days	1.2 years	1.7 years	1.4 years	Repeated changes in formula to help growth were stopped when it became apparent that patients with KARS have progressive microcephaly and failure to thrive. Genetic results utilized for prenatal genetic testing on subsequent pregnancy

C4	3 months	1.8 years	2.1 years	1.8 years	Parental anxiety reduced; parents were able to change medications

**Table 4:** Cost per patient diagnostic investigation beyond Tier 1. The year the test was ordered is listed along with the hospital charge per investigation.

Hospital for Sick Children Tier	Category of test	Case 1	Charges Case 1	Case 2	Charges Case 2	Case 3	Charges Case 3	Case 4	Charges Case 4
Tier 2	Lab/metabolite	Batten DNA testing (2009)	$817	Lysosomal enzymes (2007)	$1247	Vitamin B12 assay (2014)	$127		
	Chromosomal testing					Karyotype (2014)	$1110		
						Chromosome breakage studies (2014)	$766		
	Single gene test	CDKL5 sequencing (2009)	$6757	Angelman (2008)	$2210			Prader Willi and Angelman molecular analysis (2013)	$650
		SCN1A gene screen (2009)	$3050						
		Rett syndrome amplification (2009)	$2927						
		XLMR9 (2009)	$6757	SLC9A6 (2009)	$6757	Angelman methylation assay	$650		
	Gene panels	Lysosomal panel testing (2009)	$1247	Mito met chip (2008)	$3200	Infantile epilepsy panel (2014)	$5048	Dual genome panel (2014)	$3200
		ATP7A comprehensive panel (2013)	$6430			Microcephaly sequencing panel (2015)	$6025	Early infantile epilepsy panel (2015)	$4500
		Infantile epilepsy panel (2013)	$5048					Peroxisomal panel (2014)	$665

Tier 3	Biopsy			Thigh and sural nerve biopsy (2011)	$9558				
				Skin biopsy (2011)	$379				

*Total additional cost*			*$35,483*		*$23,351*		*$13,726*		*$9,015*
